# Mental health services in Norway, 2023

**DOI:** 10.1192/bji.2023.25

**Published:** 2023-11

**Authors:** Solveig Klæbo Reitan, Lars Lien

**Affiliations:** 1 Professor, Department of Mental Health, Faculty of Medicine and Health Sciences, Norwegian University of Science and Technology, Trondheim, Norway; 2 Professor, Faculty of Social and Health Sciences, Section for Mental Health and Rehabilitation, Inland Norway University of Applied Sciences, Elverum, Norway. Email: lars.lien@inn.no

**Keywords:** Community mental health teams, cost-effectiveness, health economics, history of psychiatry, human rights

## Abstract

Norway has, according to the World Health Organization, more psychiatrists engaged in public health services per head of population than any other country, and the proportionate numbers of psychologists and others engaged in mental healthcare are also among the world's highest. Approximately 10% of Norway's gross domestic product is spent on health, expenditure per capita that is the fourth highest internationally. We discuss how this wealth of expertise translates into the delivery of services to the public.

According to the World Health Organization, Norway possesses more psychiatrists in public health services, per head of population, than any other country.^1^ The per capita proportions of psychologists and other healthcare personnel occupied in public mental healthcare are also among the world's highest. approximately 10% of Norway's gross domestic product (GDP) is spent on health, expenditure in terms of GDP per capita that is the fourth highest internationally (figures for 2023).^2^ Most health services are public and free of charge for inhabitants (patients pay a small amount, not exceeding €300 per year). The number of private health services is relatively low. Dental health, general ophthalmological services and hearing aids are not financed from public funds, although this is controversial because it may give rise to inequality in healthcare. Other social services are widely available. Social disparities in levels of salary, access to education and access to healthcare are small. The standard of living is generally good, and 35% of the population have been educated at further education college or university level.^3^ All education, including university tuition, is free of charge. Nevertheless, Norway itself has educated only 50% of the country's medical doctors, although the proportion of other healthcare professionals who are Norwegian by birth is higher.

Norway is a constitutional monarchy: its parliament (*Stortinget*) decides new laws; a government with a parliamentary basis executes political decisions; and the legal system interprets and enforce the laws.

## The nation's health

Infant mortality in Norway is 3/1000 (reduced from 9/1000 in 1990). Life expectancy at birth is 80 years for men and 84 years for women. The usual age at retirement is 67 years. Like many other European countries, Norway is facing a relatively steep increase in the proportion of its population who are elderly, not working and who are at a high risk of morbidity, including mental health problems. The rate of suicide 12.4/100 000 population, which is higher than the European average. It has been stable in recent years despite campaigns to reduce it further, although it was higher in the past (16.4/100 000 in 1990).^4^

The prevalence of severe psychiatric disorders is very similar to figures from other wealthy industrialised countries. However, there are currently concerns that increasing numbers of young people are reporting poor mental health. This phenomenon could be related to both increased individualism in Norwegian society, which leaves some individuals feeling lonely, and the impact of young people's obsession with social media. It may also, paradoxically, be a consequence of the wide range of opportunities open to young people in this country, who can easily take advantage of educational and other choices in life. There are societal concerns that despite the fact that many young people have opportunities to create a meaningful and happy life, there will always be someone who is left behind and is unable to take advantage of these possibilities. Concerns have been expressed that although these young people may be mentally distressed, it would not be appropriate to medicalise their condition. There is a risk that health workers, health leaders and politicians confuse such distress with severe mental disorders. In our opinion, the most important task for psychiatrists in Norway is to care for the most severely mentally ill people.

## Population demographics and healthcare

The total population of Norway is about 5.5 million, approximately 1.2 million (22%) of whom are under 18 years of age. Most of this population is concentrated in and around major cities in the south of the country, including Oslo (0.7 million), Bergen (0.3 million) and Stavanger (0.14 million). In the middle of Norway is Trondheim (0.21 million). The four universities with medical schools are in Tromsø, Trondheim, Bergen and Oslo. Nurses and other healthcare personnel are educated at many more university colleges. Large areas of the country are sparsely populated, with small towns and villages. A scattered population exists throughout the north of the country, which is over 1000 miles in length from north to south. This widely dispersed population creates challenges for the provision of equitable health services to all the country's inhabitants. The predominant Norwegian political ideology has always been and still is based on social democratic ideas of equality in health, social services and education. This is in line with a country whose culture and tradition over the past centuries has always lacked a powerful local upper class. Because its population is widely dispersed, and sparse, living under extreme climatic conditions forced everyone to collaborate. These factors have led to a widespread distribution of healthcare services.

Nationwide, primary care is provided to all 356 municipalities by nurses, social workers and psychologists, together with general practitioners (GPs) (family doctors). Specialised healthcare is organised into four main regional health trusts. These trusts organise hospital care, as well as 77 district psychiatric centres. Most psychiatrists work in specialised healthcare centres (hospitals or district psychiatric centres), where they constitute a minority of the staff, compared with nurses, social workers and other health professionals. A relatively large number of clinical psychologists are employed in secondary specialised healthcare.^5^ Norwegian legislation and tradition do not recognise a medical hegemony. Psychologists have equal rights to psychiatrists regarding leadership, clinical examination, choice of treatment, psychological treatments and decisions regarding coercion for the provision of treatment, although medically trained psychiatrists are still the only profession that can prescribe certain medications. This situation has caused some disquiet to the Norwegian Psychiatric Association. Although it welcomes the fact that clinical psychologists complement the healthcare team for people with mental illness, they may not be adequately trained to provide an assessment of the somatic health of the patients, nor be competent to undertake a comprehensive psychiatric evaluation and devise a treatment plan for those with severe mental disorders.

## History of mental healthcare, laws and regulations

Norway passed its first Law on the Treatment and Care of the Insane in 1848. It declared that the responsibility for the care of the insane was the government's. A Control Commission was established to inspect the asylums and the private residences that lodged people with a mental illness and to assess patients’ legal rights. The law stated that isolation and restraint should be kept to a minimum and be documented in the establishment's records. This law underwent only minor amendment until it was replaced by the 1961 Law on Mental Healthcare. The major change was that the new law introduced the possibility of involuntary admission for an observation period that could last up to 3 weeks if there was doubt about the mental state of the patient, that is, that the patient might have a psychotic disorder. In 2001 this law was revised and the maximum observation period was reduced to 10 days. The revised law allows more use of compulsory treatment of out-patients, reducing the need for hospital beds. It was intended to improve patients’ quality of life by allowing them to stay in their own homes while they were being treated. Under the terms of Norwegian legislation, an external medical doctor must evaluate any patient who is being considered for compulsory admission before the hospital psychiatrist or psychologist can implement coercive treatment. The patient can appeal to a local control commission, then to the main courts and up to the Supreme Court to review the conditions under which they were detained. A patient who was admitted voluntarily cannot be converted to an involuntary admission.

The last main revision of the law on involuntary admissions for psychiatric care (effective from 2017) stated that no one should be admitted or treated by coercion if they are competent to consent. The number of coercively admitted patients in Norway in 2022 was 5500, making up 2.2% of the more than 250 000 individuals given treatment in specialised mental healthcare in Norway annually.^6^ There are persistent campaigns for a reduction in the use of coercive admission for treatment. A debate about the relative importance of balancing human rights and autonomy against an obligation to care for those not able to care for themselves has been long-lasting, together with need to protect society from patients who could constitute a risk to others.

The mental asylums that had been established between 1865 and 1920 have gradually been replaced by smaller hospitals and psychiatric wards in general hospitals. From the 1980s decentralisation and deinstitutionalisation have taken place. There has been a dramatic decline in the number of hospital beds and far greater provision of out-patient services. In 1998 the government launched a campaign to increase mental health services during the period 1999–2006, which was termed the Escalation Plan for Mental Health.^7^ The aim was to provide more resources to support mental healthcare. District psychiatric centres should be established and there should be a focus on decentralised services. Every Minister of Health since then has promised to provide more resources to mental healthcare and launched campaigns on the issue.

Nevertheless, in 2023 there is still a concern that many people with mental disorders do not get the appropriate treatment. All hospital trusts report that they are struggling to recruit and retain psychiatrists, and many psychiatrists say they have considered quitting because of poor working conditions. This unfortunate situation means that the provision of mental healthcare in Norway is under constant debate among professionals, leaders, politicians, journalists and user organisations. A lack of resources is only one factor that has led to widespread dissatisfaction with services. The general population is more aware of mental health issues, and campaigns aimed at improving knowledge about mental health have been comprehensive in recent years. There may have been a change in attitudes, resulting in people seeking help from psychiatric services for painful events in life, although those services were designed to manage more severe, and less prevalent, psychiatric illnesses.

## Today's mental healthcare system: economy and personnel

As described above, the design and management of the mental healthcare system in Norway is under constant debate. Today, 250 000 inhabitants get services from specialised mental healthcare annually. Most of these are treated as out-patients in ordinary out-patient clinics, and the number of out-patient consultations is increasing. In line with other Western countries, the number of in-patient beds has been reduced, and care in the community is the prevalent ideology, as it is elsewhere. However, over the past 20 years beds for psychiatric in-patients have been provided in district psychiatric centres. In municipal health centres there has been an extensive development of specially adjusted housing for patients who need admission. Healthcare and social staff are available up to 24 h a day 7 days a week. Patients have their own apartment with immediate access to staff if needed. As these services are organised differently around the country, a direct comparison with the number of ‘beds’ previously provided is not easy. Municipalities have greatly increased the number of psychiatric nurses, psychologists and social workers. In Norway, as in the rest of the world, better opportunities for treatment, recovery, participation in society and care are available nowadays.

One major national investment in recent years has been the development of assertive community treatment (ACT) teams and the related flexible ACT (FACT) teams. Their establishment is still ongoing and has extended to include teams specialising in forensic and youth FACT. Although there is already extensive research on FACT from other countries, it is still an ambition to conduct research on the effects of FACT in different Norwegian settings. Another field of investment is digital mental healthcare in different forms. This has of course been motivated in part by the need to respond to healthcare needs during the 2020 COVID-19 pandemic. However, because of the demographics of the country, which has a widespread rural population, as well as a high level of education and universal access to the internet, digitalisation of all health services has been a focus for years. Digitalised psychotherapy, telemedical consultations and supervision are under extensive development and are influencing the provision of mental healthcare.

One challenge Norwegian psychiatrists face regularly is the attitude of the antipsychiatry movement. As there is generally low respect for professional authorities in Norway, a psychiatrist who claims to provide the most effective and efficient treatment is not necessarily believed. This attitude is in stark contrast to the respect a doctor in somatic medicine receives, because his or her patients are generally willingly accept their doctor's advice. Antipsychiatry attitudes affect not only working conditions for psychiatrists, but also the priority given to their resources. Since 2016 all regional health trusts have been obliged to offer ‘treatment without medication’ for psychotic and other severe psychiatric illnesses. This shows the strong impact political decisions have had on working conditions for psychiatrists as well as the priority given to their resources. There is some research on these medicine-free services going on and we are at present waiting for results. As may be imagined, such conditions are having a negative influence on the job satisfaction of Norwegian psychiatrists.

## Training in psychiatry

Although education about mental illness and substance use problems in general medical training has improved in recent years, there is still a need to keep educators focused on the subject to ensure a good level of knowledge among GPs as well as all other doctors. After taking their final medical exams, Norwegian doctors go through an 18-month internship, which includes 6 months as a GP and 12 months in hospital specialties. In recent years, the 12 months they spend working in a hospital are increasingly likely to include a period of 4 months in a psychiatric department. This improves quality of psychiatric knowledge. Subsequently, the training period for a resident to become a specialist in psychiatry (adult psychiatry) is 5 years. That period includes clinical training in most fields (acute, long-term/rehabilitation, out-patient, etc.) and educational (theoretical) programmes covering areas such as psychotherapy, service models, the patient–therapist relationship, leadership and legislation. One year of residency may be in another relevant medical specialty. Psychotherapy education (practice and supervision) requires a minimum of 3 years of residency: 2 years of psychodynamic training, focusing mainly on the role of a therapist and the patient–therapist relationship, and a further year focusing on the treatment of patients with either psychodynamic, cognitive or group analytic therapy. This requires a minimum of 110 h of supervision in addition to theory and patient therapy. Many psychiatrists go on to further training in one or more forms of psychotherapy. Norway, like most other Western countries, has a separate specialty in child and adolescent psychiatry. In 2012 Norwegian health authorities decided to establish a medical specialty in substance use and dependency (SUD) medicine. This field overlaps with psychiatry and there are many doctors among the specialists in SUD who also are psychiatrists.

General regulations for post-graduate specialty training are organised by the national health authorities, but regional health trusts are responsible for the detailed content, including administration, leadership and research. In Norway, universities do not have any responsibility for training doctors after they leave medical school. Instead, regional and local health trusts have the responsibility. Ensuring the quality of psychiatric training in Norway is the responsibility of the Norwegian Psychiatric Association (NPA). Most psychiatrists and residents are members of the NPA, which is part of the Norwegian Medical Association (NMA). The NMA is responsible for almost 100% of all doctors in Norway as both a professional organisation and a union. The NPA is concerned with professional conditions and professional development, and the general conditions and structure of mental health services. It has specialist sections on preventive psychiatry, biological psychiatry, psychotherapy, forensic psychiatry, consultation-liaison psychiatry, old age psychiatry, emergency psychiatry, quality issues, basic problems in science and psychiatry, resident education, etc. The association and its sections work in close collaboration with the NMA, local health authorities and other professional organisations (among which is the Norwegian Psychologists’ Association) as well as several user organisations.

The NPA takes part in public and political debates about developing and changing mental health services. The restructuring and decentralisation of psychiatric services, together with legislative changes in leadership, have greatly influenced the role and function of psychiatrists. They no longer have automatic responsibility for administrative and clinical decisions because psychologists can have equivalent leadership positions. This situation has led to an extensive discussion of quality issues, indicators, diagnostic guidelines, treatment programmes and quality assurance.

Universities and university colleges, together with the hospital trusts, support a vast range of research activities in mental illness and substance use. Funding organisations nationally and internationally support such research, which has increased dramatically during the past decade. Today, most areas of psychiatry are covered by clinical, epidemiological and basic research. Research advantages include the fact that Norway has a stable population that is, in sociodemographic terms, relatively homogeneous. Patients use a limited number of hospitals and other health services, and the relatively small units serve populations locally. There is close collaboration between these clinics and academia. The low density of the Norwegian population is a challenge, so working with colleagues nationally and internationally is highly appreciated.

## Conclusions

Norway is well-supplied with personnel and resources in healthcare, although demands on those services will always exceed the resources available. Organisations and services are constantly being developed and updated services and treatments are being implemented. The field of mental health is frequently under debate in the media and among politicians. Research and education in the medical sciences are increasingly focused on mental illness and substance use, although still these fields strive to compete with somatic medicine for resources. The country's demography, as well as political will, challenges the aim to offer mental health services all over the country. Concerns have been expressed about the erosion of professional psychiatric culture because psychiatrists are spread thinly across a wide geographical area. Norwegian tradition encourages a lack of professional hierarchy, a beautiful philosophy in some ways, but it presents a challenge to psychiatrists, who are trained to a level of medical competency and up until recently have been expected to take responsibility for demanding decisions and actions. These last two factors may be among those influencing Norwegian medical graduates away from a career in psychiatry, potentially weakening the field. The National Psychiatric Association is aware of this urgent issue and is trying to find a solution.

## Data Availability

Data availability is not applicable to this article as no new data were created or analysed in this study.
